# Associations Between Shift Work and Insulin Resistance Markers in 53,053 Spanish Workers: A Sex-Stratified Cross-Sectional Analysis Using TyG, TyG-BMI, METS-IR, and SPISE-IR Indices

**DOI:** 10.3390/jcm14134604

**Published:** 2025-06-29

**Authors:** Javier Tosoratto, Pedro Juan Tárraga López, Ángel Arturo López-González, Carla Busquets-Cortes, Joan Obrador de Hevia, José Ignacio Ramirez-Manent

**Affiliations:** 1 ADEMA-Health Group, University Institute for Research in Health Sciences (IUNICS), 07010 Palma, Balearic Islands, Spain; javierlucas.tosoratto@hsll.es (J.T.); c.busquets@eua.edu.es (C.B.-C.); j.obrador@eua.edu.es (J.O.d.H.); joseignacio.ramirez@ibsalut.es (J.I.R.-M.); 2 Faculty of Medicine, University of Castilla La Mancha (UCLM), 02008 Albacete, Castilla La Mancha, Spain; pjtarraga@sescam.jccm.es; 3 Health Service of Castilla La Mancha (SESCAM), 02008 Albacete, Castilla La Mancha, Spain; 4 Faculty of Dentistry, University School ADEMA, 07010 Palma, Balearic Islands, Spain; 5 Institut d’Investigació Sanitària de les Illes Balears (IDISBA), Balearic Islands Health Research Institute Foundation, 07010 Palma, Balearic Islands, Spain; 6 Balearic Islands Health Service, 07010 Palma, Balearic Islands, Spain; 7 Faculty of Medicine, University of the Balearic Islands, 07010 Palma, Balearic Islands, Spain

**Keywords:** shift work, insulin resistance, TyG index, METS-IR, SPISE, lifestyle factors

## Abstract

**Background/Objective**: Shift work has been increasingly associated with adverse metabolic outcomes, including insulin resistance (IR), a key contributor to cardiometabolic diseases. However, few large-scale studies have explored the association between shift work and validated IR indices across sociodemographic and lifestyle variables, stratified by sex. To investigate the relationship between shift work and four surrogate markers of insulin resistance—TyG índex, TyG-BMI, METS-IR (Metabolic score for insulin resistance), and SPISE-IR (Single-Point insulin Sensitivity estimator)—in a large Spanish working population and to assess how sociodemographic and behavioral factors modify these associations. **Methods**: This cross-sectional study included 53,053 employed adults (31,753 men and 21,300 women) across various regions and labor sectors in Spain. Participants were classified as shift or non-shift workers based on their work schedules. Data were collected during routine occupational health assessments and included anthropometric, clinical, biochemical, and behavioral variables. IR indices were calculated using standard formulas. Statistical analyses included *t*-tests, chi-square tests, and multivariable logistic regression, with stratification by sex. **Results**: Shift workers exhibited significantly higher levels of TyG, TyG-BMI, and METS-IR, and lower SPISE values compared to non-shift workers (all *p* < 0.001). These differences persisted after stratification by sex, age, education, and lifestyle factors. Logistic regression analyses confirmed that shift work independently predicted high IR risk across all indices, with adjusted ORs ranging from 1.49 to 1.89. Physical inactivity, low adherence to the Mediterranean diet, and alcohol consumption were the strongest modifiable predictors. Men exhibited a higher burden of IR than women across all indices. **Conclusions**: Shift work is independently associated with elevated insulin resistance in both men and women. These findings emphasize the importance of workplace health interventions targeting physical activity, diet, and substance use, particularly in populations engaged in non-standard work schedules.

## 1. Introduction

The global workforce has undergone a profound transformation in recent decades, with a significant rise in non-traditional working hours. Shift work, encompassing evening, night, rotating, and irregular schedules, has become increasingly prevalent, particularly in industrialized nations. While essential for maintaining continuous services, this work pattern disrupts the endogenous circadian system, adversely affecting metabolic regulation and overall health [1,2]. Epidemiological evidence has consistently linked shift work with increased risk of obesity, type 2 diabetes mellitus (T2DM), metabolic syndrome, and cardiovascular disease (CVD) [3,4,5].

A central mechanism underlying these associations is insulin resistance (IR), a condition in which peripheral tissues such as muscle, liver, and adipose tissue exhibit a diminished response to insulin, prompting compensatory pancreatic hypersecretion in order to maintain normal blood glucose levels [6]. This state leads to hyperinsulinemia and constitutes a core component of both metabolic syndrome and type 2 diabetes mellitus (T2DM) [7]. T2DM is characterized by chronic hyperglycemia resulting from the combination of insulin resistance and progressive pancreatic β-cell dysfunction. It is the most common form of diabetes and is closely associated with obesity, physical inactivity, and genetic predisposition [8]. In certain contexts, it is still referred to by its former designation, non-insulin-dependent diabetes mellitus (NIDDM). Prior to the onset of T2DM, impaired glucose tolerance (IGT) may develop, defined as elevated blood glucose levels following an oral glucose load that do not meet the diagnostic thresholds for diabetes, representing a prediabetic state [9].

Insulin resistance is a complex metabolic disorder that differentially impairs the function of key organs and tissues involved in energy homeostasis. In the liver, it hinders the suppression of gluconeogenesis, leading to excessive glucose production and contributing to fasting hyperglycemia. Additionally, it promotes de novo lipogenesis, resulting in greater hepatic fat accumulation and the development of non-alcoholic fatty liver disease (NAFLD) [10]. In adipose tissue, insulin fails to effectively inhibit lipolysis, causing an increase in circulating free fatty acids, which in turn exacerbate insulin resistance in other tissues and foster a state of low-grade chronic inflammation [11]. In skeletal muscle—the primary site for insulin-stimulated glucose uptake—GLUT4 translocation to the plasma membrane is impaired, reducing glucose entry into cells and worsening glycemic control [12]. This coordinated metabolic dysfunction across tissues increases the risk of developing type 2 diabetes, NAFLD, polycystic ovary syndrome, atherosclerosis, and cardiovascular diseases [13,14]. Moreover, recent studies suggest that insulin resistance may also affect endothelial, renal, and cerebral function, thereby broadening its systemic impact [15,16].

While direct measurement of insulin sensitivity via hyperinsulinemic-euglycemic clamp remains the gold standard, its cost and technical complexity preclude routine use in large-scale studies. As a result, several surrogate indices have been developed and validated for use in epidemiological and clinical settings.

Among the most widely accepted surrogate markers are the triglyceride-glucose index (TyG), its variant incorporating body mass index (TyG-BMI), the metabolic score for insulin resistance (METS-IR), and the single point insulin sensitivity estimator (SPISE). The SPISE index is a valuable tool in epidemiological studies for detecting insulin resistance, particularly in settings where invasive or costly methods are not feasible. It relies on easily obtainable parameters such as HDL cholesterol, triglycerides, and BMI and has shown good correlation with reference methods. Its simplicity and reproducibility make it an effective marker for large-scale screening of insulin sensitivity [17].

These indices offer cost-effective and reproducible alternatives for detecting insulin resistance and predicting related outcomes [18,19,20,21]. Recent studies have validated these indices across diverse populations, highlighting their utility in both clinical and occupational health contexts [22,23].

Despite this progress, relatively few studies have explored how shift work relates to these IR indices in a sex-disaggregated manner, nor how sociodemographic, educational, and lifestyle factors moderate these associations. Gender differences in hormonal regulation, body composition, and occupational roles may differentially influence the metabolic consequences of shift work [24,25]. Therefore, it is essential to conduct sex-stratified analyses, as physiological and hormonal differences between men and women affect body fat distribution, lipid metabolism, and insulin sensitivity. Ignoring these differences may lead to misinterpretation of the prevalence and determinants of insulin resistance, as well as underestimation of sex-specific cardiometabolic risk [26]. In addition, behaviors often linked to shift work—such as smoking, low physical activity, poor dietary patterns, and alcohol use—can exacerbate metabolic disturbances [27,28].

This study aims to examine the associations between shift work and four surrogate markers of insulin resistance (TyG, TyG-BMI, METS-IR, SPISE) in a large cohort of over 53,000 Spanish workers, stratified by sex. We further investigate how age, social class, educational level, physical activity, dietary patterns, smoking, and alcohol consumption relate to these indices, and whether these factors modify the observed associations.

## 2. Materials and Methods

### 2.1. Study Design and Population

This cross-sectional, observational study included a total of 53,053 employed adults from a broad spectrum of economic sectors and geographic regions throughout Spain. Participants were recruited during routine occupational health examinations conducted between January 2021 and June 2022. The cohort comprised 31,753 men (including 17,527 shift workers) and 21,300 women (11,281 of whom performed shift work).

Eligibility criteria were as follows:

(1) age between 18 and 69 years;
(2) active employment under a formal labor contract with a participating company;
(3) signed informed consent to participate; and
(4) explicit authorization for use of anonymized data for epidemiological purposes.

**Figure 1:**
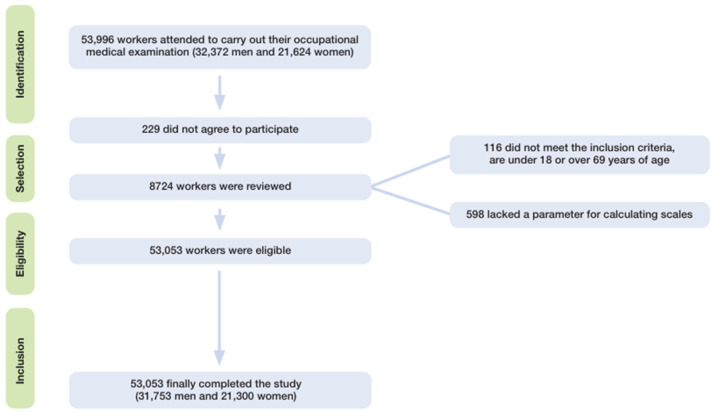
Displays the data from the workers’ flowchart after applying the inclusion criteria.

The participant selection process and final analytic sample distribution are detailed in Figure 1.

### 2.2. Data Collection Procedures

Data were collected by trained personnel from occupational health units using standardized protocols. Comprehensive medical history interviews were conducted to gather information on sociodemographic attributes (age, sex, educational attainment, and occupational-based social class) and health-related behaviors, including smoking status, alcohol consumption, physical activity, and adherence to the Mediterranean dietary pattern.

### 2.3. Anthropometric and Clinical Assessments

Anthropometric measurements—height, weight, and waist circumference—were taken in accordance with international standards set by the International Society for the Advancement of Kinanthropometry (ISAK) [29]. Participants were assessed in light clothing and without shoes. Body weight and height were recorded using a SECA 700 stadiometer and scale (SECA, Chino, CA, USA), and waist circumference was determined at the midpoint between the lower margin of the last palpable rib and the top of the iliac crest using a flexible, non-stretchable SECA tape (SECA, Chino, CA, USA).

Blood pressure was recorded using an automated oscillometric monitor OMROM-M3 device (OMRON, Osaka, Japan) after participants were seated quietly for at least 10 min. Three readings were taken at one-minute intervals, and the average was used for analysis. Hypertension was defined as systolic blood pressure ≥ 140 mmHg, diastolic blood pressure ≥ 90 mmHg, or use of antihypertensive medication.

### 2.4. Biochemical Analyses

Venous blood samples were collected following a minimum fasting period of 12 h. Samples were transported under controlled conditions and analyzed within 72 h in certified laboratories. Fasting glucose, triglycerides, and total cholesterol concentrations were determined via enzymatic colorimetric assays. HDL-cholesterol (high-density lipoproteins-cholesterol) was measured using selective precipitation methods, while LDL-cholesterol (low-density lipoproteins-cholesterol) was estimated using the Friedewald equation [30] when triglyceride levels were <400 mg/dL; otherwise, direct LDL assays were employed. All measurements were expressed in mg/dL. Dyslipidemia and hyperglycemia were defined using clinical cutoffs or based on current pharmacological treatment.

### 2.5. Insulin Resistance Risk Scales

- **TyG index** [31]. TyG = LN (triglycerides × glycaemia/2) is considered high risk at 8.5.
- **TyG-BMI** [32] TyG-BMI = TyG × BMI is considered high risk at 185.
- **Metabolic score for insulin resistance (METS-IR)** [33]. METS-IR = Ln(2 × glucose) + triglycerides × BMI)/(Ln(HDL-c). High values are defined as 50 and above.
- **Single-Point insulin Sensitivity estimator (SPISE-IR)**. SPISE = (=600 × HDL0.185/triglycerides 0.2 × BMI1.338). SPISE-IR [34] = 10/SPISE is considered high risk at 1.51.

BMI = body mass index

### 2.6. Operational Definitions and Variable Classification

- **Sex** was recorded as male or female.
- **Age** was calculated from birthdate and date of examination.
- **Educational level** was categorized as primary (elementary), secondary (high school), or university education.
- **Social class** classification was based on the Spanish Society of Epidemiology guidelines, using the 2011 National Classification of Occupations (CNO-11) [35], grouping individuals into the following:
  ○ Class I includes individuals engaged in highly skilled occupations that require a university degree or equivalent, often involving managerial responsibilities or specialized intellectual functions. These roles are characterized by a high level of job autonomy, strategic decision-making, and oversight of human or material resources. This category also encompasses creative professions that demand specific skills not necessarily obtained through formal academic education, such as professional athletes and recognized artists. This group includes professionals, executives, athletes, and artists with higher education.
  ○ Class II comprises individuals performing occupations that require intermediate technical or professional qualifications, typically obtained through vocational training at the intermediate or advanced level, and in some cases, through short-cycle higher education. This class also includes skilled self-employed workers, such as small business owners or freelancers who do not employ others but carry out complex or specialized tasks. It encompasses technicians, intermediate professions, and qualified self-employed workers.
  ○ Class III includes both skilled and unskilled manual workers, whose occupations generally do not require higher academic education. These jobs are characterized by physical or repetitive tasks, often performed under direct supervision, with low autonomy, and in settings involving exposure to physical or ergonomic hazards. This group typically faces less job stability and poorer working and health conditions. It includes manual laborers and occupations requiring lower qualifications.
- **Smoking status** was defined as current smoking or cessation within the past 12 months [36].
- **Adherence to the Mediterranean diet** was evaluated using a validated 14-item questionnaire. A score ≥ 9 indicated high adherence [37].
- **Physical activity** levels were self-reported via the short-form International Physical Activity Questionnaire (IPAQ), assessing activity during the previous seven days [38].
- **Alcohol intake** was quantified in standard drink units (SDUs), where 1 SDU corresponds to 10 g of ethanol. Thresholds for high-risk consumption were >35 SDUs/week in men and >20 SDUs/week in women, consistent with national guidelines [39].
- **Shift work** was defined as any regular work schedule that deviated from the standard daytime hours (typically 9 am to 5 pm), including rotating shifts, evening shifts, night work, and split shifts [40].

### 2.7. Statistical Analyses

Continuous variables were summarized as means and standard deviations, and intergroup comparisons were conducted using Student’s *t*-test. Categorical variables were reported as frequencies and percentages, with differences assessed using the Chi-square test. To examine associations between shift work and insulin resistance risk (based on elevated TyG, TyG-BMI, METS-IR, and low SPISE scores), multivariable logistic regression models were employed. Odds ratios (ORs) with 95% confidence intervals (CIs) were calculated. Model fit was evaluated using the Hosmer–Lemeshow goodness-of-fit test.

Potential confounding variables were assessed through stratified analyses; no significant effect modification was observed. Statistical analyses were performed using IBM SPSS Statistics software (version 28.0, IBM Corp., Armonk, NY, USA), with a two-sided *p*-value of <0.05 considered indicative of statistical significance.

## 3. Results

Table 1 describes the demographic, anthropometric, and lifestyle characteristics of 53,053 workers (24,245 women and 28,808 men). Shift workers showed significantly worse metabolic profiles across both sexes. Male shift workers had higher weight (84.5 vs. 81.5 kg), waist circumference (90.8 vs. 89.5 cm), blood pressure, total cholesterol, LDL-C, triglycerides, and fasting glucose compared to non-shift males (all *p* < 0.001). Similar trends were observed in women, with shift workers showing notably higher weight (63.6 vs. 68.8 kg), waist circumference (77.6 vs. 74.7 cm), and systolic blood pressure (116.1 vs. 114.8 mmHg). Shift workers were more likely to be physically inactive (67.9% of men and 60.7% of 171 women), adhere less to Mediterranean diet (5% vs 41.8% of men and 36.9% vs. 58% of women), and consume more alcohol (36.8% vs. 29.6% of men and 16.5% vs. 14.7% of women), all known contributors 172 to IR development [41].

Table 2 shows clear trends of worsening IR indices (higher TyG, TyG-BMI, METS-IR; lower SPISE) with advancing age and lower social position. Male shift workers exhibited consistently higher values across all age and social groups. For instance, in the 50–59 age group, METS-IR was 47.0 in shift workers vs. 46.4 in non-shift workers. SPISE-IR scores declined with age and were worse in shift workers. Physical inactivity and poor diet markedly increased TyG-BMI (261.5 in inactive shift workers 183 vs. 197.2 in active ones) and increased SPISE-IR (2.3 vs. 1.4), confirming previous findings on behavioral 184 contributors to IR [42].

Similar patterns were observed in women. Across all age groups, shift workers had higher TyG, TyG-BMI, and METS-IR, and lower SPISE scores. For example, METS-IR in women aged 60–69 was 44.5 in shift workers vs. 43.4 in non-shift workers. Social gradients were evident: SPISE-IR scores were higher among those with elementary education and high school education than those with university degrees. Women adhering to the Mediterranean diet had lower TyG-BMI (178.3 vs. 240.1) and lower SPISE-IR (1.2 vs. 1.9), emphasizing the protective role of diet [43] (Table 3).

Table 4 presents percentages of men exceeding high-risk thresholds for each IR index. The burden of IR was higher among shift workers. For instance, among male shift workers aged 60–69, 58.5% had high TyG, 53.4% had high TyG-BMI, and 83.4% had high SPISE-IR. Physical inactivity was associated with particularly high risk, and 86.7% of inactive shift workers had high SPISE-IR. High alcohol consumption further increased the percentage of both non-shift and shift workers with higher SPISE-IR value. These findings align with the prior literature linking circadian disruption, poor sleep, and substance use with insulin resistance [44].

Women also show similar trends of increased IR indices in shift workers. For example, in the 50–59 age group, 55.6% of female shift workers had higher SPISE-IR versus 45.0% of non-shift workers. Physical inactivity and low education significantly increased the prevalence of IR. Notably, university-educated women had the lowest risk. Alcohol consumption was a major determinant: 80.7% of female shift workers who consumed alcohol had higher SPISE-IR, underscoring its role as a metabolic disruptor [45] (Table 5).

Table 6 shows that compared to non-shift workers, shift workers have an OR value (with 95% CI) of 1.89 for high TG, an OR value (with 95% CI) of 1.49 for high TyG-BMI, and an OR value (with 95% CI) of 1.83 for high SPISE-IR. Other strong predictors included male sex (e.g., OR = 3.54 for higher SPISE-IR), physical inactivity (OR = 16.30 for higher SPISE-IR), non-adherence to the Mediterranean diet (OR = 2.87 for higher SPISE-IR), and alcohol consumption (OR = 4.62 for higher SPISE-IR). These results reinforce the multifactorial etiology of IR and highlight modifiable targets for intervention [46] (Table 6).

## 4. Discussion

This large, cross-sectional study of more than 53,000 Spanish workers provides robust evidence that shift work is significantly associated with increased insulin resistance, as measured by four validated indices: TyG, TyG-BMI, METS-IR, and SPISE-IR. The findings remain consistent after stratification by sex, age, social class, education, and key lifestyle factors and are further reinforced by multivariate logistic regression analyses that demonstrate independently elevated odds of adverse IR profiles in shift workers.

Our findings are consistent with previous studies indicating that circadian rhythm disruption—common among individuals engaged in shift work or exposed to prolonged artificial light—is associated with the development of insulin resistance through multiple biological mechanisms. Among these, the desynchronization between the central clock (located in the suprachiasmatic nucleus) and peripheral clocks in metabolically active organs such as the liver, skeletal muscle, and adipose tissue stands out. This misalignment alters the expression of genes involved in glucose and lipid homeostasis, impairing glucose uptake, insulin secretion, and peripheral sensitivity. In addition, sleep disturbances and irregular eating patterns promote a pro-inflammatory state characterized by increased levels of cytokines such as TNF-α and IL-6, which interfere with insulin signaling. Ultimately, elevated cortisol secretion and disrupted melatonin rhythms further contribute to metabolic dysfunction and reduced insulin sensitivity [47,48].

Additionally, behavioral changes frequently associated with shift work—poor diet, sedentarism, increased alcohol consumption, and smoking—may further exacerbate insulin resistance, as observed in our cohort.

Our analysis of sex differences revealed that while both men and women experienced worse IR profiles with shift work, the absolute burden was higher in men. This may reflect underlying differences in fat distribution, hormonal modulation, and occupational exposures [49]. Men tended to exhibit higher triglyceride levels and waist circumference, factors directly contributing to elevated TyG and METS-IR scores. Conversely, women presented more favorable SPISE scores, consistent with the prior literature indicating greater insulin sensitivity in premenopausal women [50].

The strong association between low physical activity and insulin resistance across all indices is particularly noteworthy. Workers with less physical activities, regardless of sex or shift status, exhibited dramatically worse IR profiles. For instance, SPISE-IR scores were nearly 50% higher in physically inactive compared to active individuals. This finding corroborates the protective effect of physical activity on insulin signaling and glucose metabolism [51] and underscores its importance in workplace health promotion strategies.

Dietary patterns also played a significant role. Workers with low adherence to the Mediterranean diet displayed markedly elevated TyG-BMI and METS-IR scores and higher SPISE-IR values, even after adjustment for other variables. The Mediterranean diet has been consistently associated with improved metabolic profiles, lower IR, and reduced incidence of T2DM and CVD in multiple cohorts [52,53]. Our findings suggest that this dietary pattern may be particularly beneficial in shift workers, who are at elevated metabolic risk.

Dietary patterns and physical activity can also influence serum uric acid levels, which have been associated with insulin resistance. The interplay between elevated serum uric acid levels and insulin resistance (IR) has emerged as a topic of growing interest in metabolic research. Accumulated evidence suggests that hyperuricemia is not only associated with elevated fasting plasma glucose levels but may also play an active pathogenic role in glucose metabolism dysregulation. This observation is particularly relevant given the sustained global increase in the prevalence of metabolic syndrome, type 2 diabetes, and cardiovascular diseases [54].

From a pathophysiological perspective, uric acid has been proposed to induce insulin resistance through several mechanisms. These include reduced bioavailability of nitric oxide (NO)—a key molecule in insulin signaling and endothelial blood flow regulation—as well as increased mitochondrial oxidative stress and low-grade inflammation triggered by elevated urate levels, all of which appear to contribute to impaired insulin sensitivity. Conversely, IR may promote hyperuricemia by enhancing renal tubular reabsorption of sodium and uric acid, reinforcing the notion of a bidirectional relationship between these two conditions [55].

Despite this mechanistic foundation, epidemiological findings remain heterogeneous. Some longitudinal studies have suggested that elevated uric acid levels can predict the future onset of insulin resistance, while others have identified IR as an independent risk factor for subsequent hyperuricemia. This lack of consensus reflects a complex temporal dynamic, in which alterations in either condition may precede or exacerbate changes in the other, depending on the physiological context or the presence of additional risk factors [56].

The relationship between hyperuricemia and insulin resistance also presents novel therapeutic opportunities. Although urate-lowering therapies—such as xanthine oxidase inhibitors—have traditionally been used to manage gout, they may offer additional metabolic benefits in patients with hyperuricemia and insulin resistance, potentially aiding in the prevention of type 2 diabetes and its cardiometabolic complications. However, this approach requires validation through well-designed clinical trials [57]. A deeper understanding of the underlying mechanisms and the directionality of this relationship is essential to inform more effective and personalized clinical strategies for the management of metabolic disorders.

Unfortunately, serum urate was not included in the routine biochemical panel obtained during occupational health examinations in our study. We believe its inclusion would have provided valuable insights and recommend its incorporation in future investigations.

Another significant finding was the impact of alcohol consumption. While moderate alcohol intake has sometimes been associated with improved insulin sensitivity, our results indicate that alcohol consumption—especially among shift workers—is linked to worse IR profiles. This reflects binge drinking patterns or alcohol-related circadian disruptions, which have been shown to interfere with glucose metabolism [58].

Socioeconomic and educational gradients were evident across all analyses. Workers with lower educational attainment and those from lower social classes had consistently worse IR profiles, independent of shift status. These findings are consistent with broader evidence linking social disadvantage with metabolic disease through pathways of chronic stress, limited health literacy, reduced access to healthy food and recreational facilities, and higher rates of unhealthy behaviors [59,60].

The logistic regression analyses confirmed the independent effect of shift work on insulin resistance, with ORs ranging from 1.49 (METS-IR) to 1.89 (TyG) after full adjustment. These estimates are in line with a recent meta-analysis showing that shift workers had a 1.2–1.6-fold increased risk of metabolic syndrome and IR compared to non-shift workers [61]. Importantly, the associations remained significant after controlling for confounders such as age, sex, physical activity, and dietary patterns, indicating that the effects of shift work per se—beyond associated behaviors—contribute to metabolic risk.

Nevertheless, despite having controlled for several confounding factors, it is important to consider the inherent heterogeneity of shift work, as different scheduling patterns may exert distinct physiological effects. This aspect is particularly relevant when interpreting the results, since not all shift work arrangements impact circadian rhythms and metabolic regulation in the same way [62]. In addition, unmeasured potential confounders in the present study—such as sleep quality and duration [63], chronotype (morning/evening preference), and specific occupational stress—should also be taken into account, as all of them are closely related to both metabolic health and the ability to adapt to shift work [64]. The absence of these variables may limit a comprehensive understanding of the underlying mechanisms. In this regard, future research should incorporate the systematic assessment of these factors using validated instruments, which would enhance analytical precision and provide a more complete perspective on the impact of shift work on workers’ metabolic profiles.

Our findings have important public health and occupational medicine implications. Given the growing proportion of shift workers in modern economies, targeted interventions to mitigate insulin resistance and prevent downstream metabolic diseases are urgently needed.

Strategies may include (1) promoting structured physical activity programs during or adjacent to work shifts; (2) facilitating access to healthy foods and adherence to Mediterranean-like diets in workplace cafeterias; (3) providing education on sleep hygiene, substance use, and stress management; and (4) considering organizational changes to reduce circadian disruption, such as forward-rotating shifts and limiting night work duration, in accordance with the WHO Healthy Workplace Framework and the EU Working Time Directive [65].

The implementation of workplace programs aimed at reducing insulin resistance through healthy lifestyle interventions may offer substantial economic benefits, both for employers and healthcare systems. Interventions promoting regular physical activity, balanced dietary habits, smoking cessation, alcohol moderation, adequate sleep, and stress reduction have demonstrated efficacy in improving insulin sensitivity and preventing the progression to type 2 diabetes.

From an occupational health perspective, such improvements are associated with decreased rates of absenteeism, reduced presenteeism, and enhanced overall productivity. Employees with better metabolic profiles are less likely to suffer from fatigue, mood disturbances, and comorbidities that compromise work performance. Additionally, companies that invest in preventive health strategies may experience lower healthcare costs due to a reduced burden of chronic disease, fewer medical leave episodes, and improved long-term employee retention.

The return on investment (ROI) of such programs is further strengthened by indirect gains, including a more resilient workforce and a positive organizational culture centered on well-being. These findings support the integration of metabolic health promotion into occupational health policies, particularly in populations exposed to metabolic stressors such as shift work. Policymakers and employers should consider these potential economic advantages when designing or scaling up interventions targeting insulin resistance, recognizing that the long-term savings and productivity gains may outweigh the initial implementation costs [66]. This approach requires interdisciplinary collaboration among occupational health services, primary care physicians, endocrinology specialists, and socio-health governmental policies.

It is important to note, in conclusion, that this is a cross-sectional study, and therefore our findings indicate an association between shift work and insulin resistance, rather than a causal relationship. In this context, conducting a longitudinal study on a subcohort would be valuable to assess changes in insulin resistance indices over time. In which the specific cut-off values for the SPISE-IR could also be studied, stratified by demographic groups, by age and sex. Additionally, a study incorporating objective behavioral measures and collecting data on sleep and chronotype could be designed.

### 4.1. Strengths

- **Large and diverse sample size:** With over 53,000 participants of both sexes across a wide age range and multiple occupational settings, this study has strong statistical power and external validity.
- **Comprehensive assessment of IR:** We evaluated four validated insulin resistance indices (TyG, TyG-BMI, METS-IR, and SPISE), offering a nuanced and multidimensional view of metabolic health.
- **Detailed stratification:** Stratified analyses by sex, age, social class, education, and lifestyle allow for exploration of effect modification and high-risk subgroups.
- **Real-world data:** This study reflects real occupational exposures and behaviors in a Mediterranean country, enhancing relevance for public health policy.

### 4.2. Limitations

- **Cross-sectional design:** Causality cannot be established, and reverse causation (e.g., workers with poorer health self-selecting into shift work) cannot be ruled out.
- Another important limitation arises from the use of self-administered questionnaires, as this type of tool is prone to biases such as recall bias and social desirability bias. To enhance the validity of the findings, future research should consider incorporating objective validation methods to complement the self-reported data.
- **Unmeasured confounders:** Despite extensive adjustment, residual confounding by variables such as sleep quality, chronotype, or work stress may persist.
- Another limitation of this study is that, since it was conducted in a Spanish working population, its applicability to populations with different genetic backgrounds, dietary patterns, or work cultures may be limited.
- **No direct insulin measurements:** The use of surrogate IR indices, although validated, does not replace direct measurement of insulin sensitivity.
- The inherent heterogeneity of shift work—characterized by varying scheduling patterns—and the absence of stratification in our study represent an additional limitation, as different shift schedules may elicit diverse physiological responses.
- Finally, data on potential confounders—including comorbidities and medication use—were not available and thus could not be included in the analysis.

## 5. Conclusions

This study provides strong evidence that shift work is associated with increased insulin resistance in both male and female workers, as assessed by four validated indices. The effect is compounded by adverse lifestyle behaviors and social disadvantage. Physical inactivity, poor adherence to the Mediterranean diet, and alcohol consumption emerged as key modifiable risk factors.

These findings highlight the urgent need for targeted workplace interventions and public health strategies to address metabolic risk among shift workers. Future research should employ longitudinal designs to establish causality and investigate the efficacy of mitigation strategies such as dietary counseling, scheduled physical activity, and optimized shift scheduling.

Given the growing reliance on shift-based labor in the global economy, protecting the metabolic health of this workforce is a critical priority for both clinical and policy action.

## Figures and Tables

**Table 1:** Characteristics of the study population by sex and shift work status.

	Non Shift Work	Shift Work		Non Shift Work	Shift Work	
	Men *n* = 14,226	Men *n* = 17,527		Women *n* = 10,019	Women *n* = 11,281	
	Mean (SD)	Mean (SD)	*p*-Value	Mean (SD)	Mean (SD)	*p*-Value
Age (years)	41.2 (10.9)	41.3 (10.5)	0.089	40.0 (10.5)	40.2 (10.3)	0.199
Height (cm)	173.8 (7.1)	173.7 (7.1)	0.219	161.0 (6.6)	161.2 (6.6)	0.015
Weight (kg)	81.5 (14.6)	84.5 (14.4)	<0.001	63.6 (12.8)	68.6 (12.8)	<0.001
Waist (cm)	89.5 (10.5)	90.8 (10.2)	<0.001	74.7 (9.7)	77.6 (10.9)	<0.001
Systolic BP (mmHg)	125.3 (15.7)	126.9 (16.0)	<0.001	114.8 (15.5)	116.1 (15.6)	<0.001
Diastolic BP (mmHg)	75.9 (10.7)	77.2 (11.0)	<0.001	70.3 (10.6)	71.6 (10.8)	<0.001
Total cholesterol (mg(dL)	197.3 (38.4)	201.2 (38.6)	<0.001	192.3 (36.6)	196.9 (37.3)	<0.001
HDL-cholesterol (mg/dL)	50.4 (7.8)	49.7 (7.7)	<0.001	55.0 (9.1)	54.5 (9.2)	<0.001
LDL-cholesterol (mg/dL)	120.9 (37.3)	123.8 (37.6)	<0.001	119.6 (36.9)	123.5 (37.5)	<0.001
Triglycerides (mmHg)	129.3 (93.7)	136.8 (95.5)	<0.001	87.5 (46.8)	93.6 (51.7)	<0.001
Glucose (mg/dL)	91.9 (26.4)	93.3 (26.4)	<0.001	86.6 (19.0)	87.8 (17.6)	<0.001
	**%**	**%**	***p*-Value**	**%**	**%**	***p*-Value**
18–29 years	16.4	13.8	<0.001	18.6	17.5	0.135
30–39 years	29.3	29.8		31.0	31.3	
40–49 years	29.0	31.3		29.6	30.6	
50–59 years	20.9	20.9		17.9	17.5	
60–69 years	4.4	4.2		2.9	3.1	
Social class I	6.8	8.2	<0.001	11.6	14.6	<0.001
Social class II	20.7	26.6		27.6	37.0	
Social class III	72.5	65.2		60.8	48.4	
Elementary school	69.5	63.8	<0.001	53.7	43.2	<0.001
High school	24.4	28.9		36.2	44.2	
University	6.1	7.3		10.1	12.6	
Non-smokers	67.9	66.0	<0.001	66.3	69.1	<0.001
Smokers	32.1	34.0		33.7	30.9	
Non physical activity	55.2	67.9	<0.001	40.8	60.7	<0.001
Yes physical activity	44.8	32.1		59.2	39.3	
Non Mediterranean diet	58.2	71.5		42.0	63.1	
Yes Mediterranean diet	41.8	28.5		58.0	36.9	
Non alcohol consumption	70.4	63.2	<0.001	85.3	83.5	<0.001
Yes alcohol consumption	29.6	36.8		14.7	16.5	

BP Blood pressure. HDL High density lipoprotein. LDL Low density lipoprotein. SD Standard deviation.

**Table 2:** Mean insulin resistance indices by age, social class, education, and lifestyle in men.

		Non Shift Work					Shift Work			
		TyG	TyG-BMI	METS-IR	SPISE-IR		TyG	TyG-BMI	METS-IR	SPISE-IR
Men	*n*	Mean (SD)	Mean (SD)	Mean (SD)	Mean (SD)	*n*	Mean (SD)	Mean (SD)	Mean (SD)	Mean (SD)
18–29 years	2329	8.1 (0.5)	199.7 (37.7)	36.0 (6.8)	1.4 (0.4)	2425	8.2 (0.5)	213.3 (43.8)	38.5 (8.1)	1.6 (0.5)
30–39 years	4174	8.4 (0.6)	218.6 (43.1)	39.5 (8.0)	1.6 (0.5)	5228	8.5 (0.6)	233.8 (46.0)	42.4 (8.7)	1.8 (0.5)
40–49 years	4130	8.6 (0.6)	240.0 (49.1)	43.7 (9.2)	1.8 (0.6)	5477	8.7 (0.6)	247.0 (45.4)	45.0 (8.6)	1.9 (0.5)
50–59 years	2972	8.8 (0.6)	252.5 (47.8)	46.4 (9.0)	2.0 (0.5)	3666	8.8 (0.6)	255.7 (44.4)	47.0 (8.4)	2.0 (0.5)
60–69 years	621	8.9 (0.5)	256.6 (43.2)	47.3 (8.2)	2.0 (0.5)	731	8.9 (0.5)	261.7 (41.5)	48.2 (8.0)	2.1 (0.5)
Social class I	972	8.4 (0.6)	225.2 (44.7)	41.1 (8.6)	1.7 (0.5)	1438	8.5 (0.6)	231.1 (41.1)	42.3 (8.0)	1.8 (0.5)
Social class II	2942	8.5 (0.6)	230.1 (49.2)	41.9 (9.2)	1.8 (0.5)	4669	8.6 (0.6)	238.9 (43.5)	43.4 (8.3)	1.9 (0.5)
Social class III	10,312	8.5 (0.7)	233.5 (48.8)	42.4 (9.2)	1.7 (0.5)	11,420	8.6 (0.6)	242.9 (49.0)	44.3 (9.3)	1.9 (0.5)
Elementary school	9874	8.6 (0.6)	238.3 (52.6)	43.3 (9.8)	1.8 (0.6)	11,169	8.7 (0.7)	242.8 (47.1)	44.1 (8.9)	1.9 (0.5)
High school	3478	8.6 (0.7)	229.5 (47.6)	42.0 (9.0)	1.7 (0.5)	5070	8.7 (0.6)	240.9 (47.7)	43.9 (9.1)	1.9 (0.5)
University	874	8.5 (0.6)	227.5 (44.6)	41.4 (9.0)	1.7 (0.5)	1288	8.6 (0.7)	232.5 (41.0)	42.6 (8.0)	1.8 (0.5)
Non-smokers	9656	8.4 (0.6)	219.4 (48.1)	40.0 (9.2)	1.6 (0.5)	11,567	8.5 (0.6)	238.0 (48.6)	43.6 (9.5)	1.8 (0.6)
Smokers	4570	8.5 (0.7)	235.7 (48.4)	42.8 (9.0)	1.8 (0.5)	5960	8.6 (0.6)	242.3 (46.2)	44.0 (8.6)	1.9 (0.5)
Non physical activity	7851	8.8 (0.6)	259.9 (44.7)	47.5 (8.4)	2.0 (0.5)	11,899	8.9 (0.6)	261.5 (41.6)	48.8 (7.9)	2.3 (0.5)
Yes physical activity	6375	8.1 (0.4)	194.1 (21.9)	35.0 (4.0)	1.3 (0.2)	5628	8.2 (0.4)	197.2 (21.3)	35.6 (3.8)	1.4 (0.2)
Non Mediterranean diet	8275	8.7 (0.6)	250.5 (46.2)	46.1 (8.8)	2.0 (0.5)	12,536	8.8 (0.6)	258.3 (43.0)	47.2 (8.3)	2.2 (0.5)
Yes Mediterranean diet	5951	8.0 (0.4)	194.2 (22.1)	35.2 (4.1)	1.3 (0.2)	4991	8.1 (0.4)	196.9 (21.4)	35.7 (3.9)	1.4 (0.2)
Non alcohol consumption	8996	8.3 (0.5)	204.6 (28.9)	37.1 (5.4)	1.5 (0.3)	12,332	8.4 (0.5)	209.5 (40.3)	39.1 (7.7)	1.6 (0.4)
Yes alcohol consumption	5230	8.9 (0.7)	275.0 (43.9)	49.3 (8.4)	2.0 (0.5)	5195	9.1 (0.7)	286.5 (44.3)	53.3 (8.4)	2.3 (0.5)

TyG Triglyceride glucose index. BMI Body mass index. METS-IR Metabolic score for insulin resistance. SPISE Single point insulin sensitivity.

**Table 3:** Mean IR indices by age, social class, education, and lifestyle in women.

		Non Shift Work					Shift Work			
		TyG	TyG-BMI	METS-IR	SPISE-IR		TyG	TyG-BMI	METS-IR	SPISE-IR
Women	*n*	Mean (SD)	Mean (SD)	Mean (SD)	Mean (SD)	*n*	Mean (SD)	Mean (SD)	Mean (SD)	Mean (SD)
18–29 years	1869	7.9 (0.4)	179.4 (32.8)	32.0 (5.9)	1.2 (0.3)	1975	8.0 (0.5)	200.6 (48.0)	35.8 (8.7)	1.4 (0.5)
30–39 years	3103	8.0 (0.5)	187.9 (39.9)	33.8 (7.3)	1.3 (0.4)	3530	8.1 (0.5)	208.6 (50.3)	37.5 (9.2)	1.5 (0.5)
40–49 years	2965	8.1 (0.5)	206.1 (48.0)	37.1 (8.7)	1.5 (0.5)	3450	8.2 (0.8)	221.3 (49.5)	39.8 (9.0)	1.6 (0.5)
50–59 years	1791	8.4 (0.5)	228.4 (54.1)	41.3 (9.9)	1.7 (0.6)	1974	8.5 (0.5)	237.2 (50.0)	42.7 (9.2)	1.8 (0.5)
60–69 years	291	8.5 (0.5)	241.0 (47.3)	43.4 (8.5)	1.8 (0.5)	352	8.6 (0.5)	246.5 (49.1)	44.5 (8.9)	1.9 (0.5)
Social class I	1164	8.0 (0.4)	181.3 (32.4)	32.2 (6.1)	1.2 (0.3)	1644	8.1 (0.5)	198.1 (43.4)	35.3 (8.0)	1.4 (0.4)
Social class II	2763	8.1 (0.5)	190.1 (41.1)	34.1 (7.4)	1.3 (0.4)	4175	8.1 (0.5)	209.2 (47.0)	37.6 (8.6)	1.5 (0.5)
Social class III	6092	8.2 (0.5)	208.8 (51.1)	37.6 (9.3)	1.5 (0.5)	5462	8.3 (0.5)	229.2 (53.6)	41.3 (9.7)	1.7 (0.6)
Elementary school	5377	8.1 (0.5)	208.5 (50.7)	37.7 (9.2)	1.5 (0.5)	4871	8.2 (0.5)	228.6 (52.3)	41.3 (9.5)	1.7 (0.5)
High school	3628	8.1 (0.5)	194.1 (45.0)	34.8 (8.1)	1.3 (0.4)	4984	8.2 (0.5)	212.2 (50.1)	38.1 (9.1)	1.5 (0.5)
University	1014	8.0 (0.4)	180.4 (30.1)	31.9 (5.5)	1.2 (0.3)	1426	8.1 (0.4)	196.3 (41.6)	34.9 (7.7)	1.4 (0.4)
Non-smokers	6638	8.1 (0.5)	195.2 (44.2)	35.2 (8.0)	1.3 (0.4)	7794	8.2 (0.5)	213.6 (49.2)	38.5 (9.0)	1.5 (0.5)
Smokers	3381	8.1 (0.5)	203.2 (49.4)	36.5 (9.0)	1.4 (0.5)	3487	8.2 (0.5)	218.9 (52.1)	39.3 (9.5)	1.6 (0.5)
Non physical activity	4090	8.4 (0.5)	237.1 (52.4)	42.9 (9.4)	1.7 (0.5)	6842	8.5 (0.5)	253.5 (48.0)	44.9 (8.7)	1.9 (0.5)
Yes physical activity	5929	7.9 (0.4)	175.2 (20.2)	31.3 (3.7)	1.2 (0.2)	4439	8.1 (0.4)	176.8 (20.8)	31.6 (3.8)	1.2 (0.2)
Non Mediterranean diet	4206	8.3 (0.5)	233.2 (54.6)	42.0 (9.9)	1.7 (0.6)	7115	8.5 (0.5)	240.1 (49.8)	44.2 (9.1)	1.9 (0.5)
Yes Mediterranean diet	5813	7.9 (0.4)	176.8 (21.3)	31.7 (3.9)	1.2 (0.2)	4166	7.9 (0.4)	178.3 (21.9)	31.9 (4.0)	1.2 (0.2)
Non alcohol consumption	8361	8.0 (0.4)	186.3 (31.5)	33.5 (5.8)	1.3 (0.3)	9619	8.1 (0.5)	189.3 (32.0)	34.3 (8.2)	1.5 (0.5)
Yes alcohol consumption	1658	8.6 (0.6)	272.1 (52.0)	48.9 (9.6)	2.1 (0.6)	1662	8.9 (0.6)	295.7 (53.8)	51.3 (9.9)	2.6 (0.6)

TyG Triglyceride glucose index. BMI Body mass index. METS-IR Metabolic score for insulin resistance. SPISE Single point insulin sensitivity.

**Table 4:** Prevalence of high-risk IR categories in men by sociodemographic and lifestyle factors.

		Non Shift Work					Shift Work			
		TyG High	TyG-BMI High	METS-IR High	SPISE-IR High		TyG High	TyG-BMI High	METS-IR High	SPISE-IR High
Men	*n*	%	%	%	%	*n*	%	%	%	%
18–29 years	2329	9.0	8.1	4.6	20.4	2425	12.7	16.1	9.3	34.4
30–39 years	4174	18.7	15.7	9.9	37.3	5228	24.7	27.0	16.7	54.9
40–49 years	4130	35.2	32.6	21.3	59.4	5477	36.5	38.3	24.1	69.4
50–59 years	2972	46.5	45.0	31.3	72.4	3666	49.3	47.4	32.8	77.4
60–69 years	621	54.8	45.9	33.5	77.8	731	58.5	53.4	37.9	83.4
Social class I	972	21.6	23.1	15.7	44.5	1438	25.5	24.3	16.3	55.8
Social class II	2942	29.8	26.3	17.5	50.4	4669	31.7	31.4	18.8	62.8
Social class III	10,312	31.2	29.9	19.7	51.9	11,420	33.5	36.8	24.4	63.2
Elementary school	9874	35.9	33.4	22.6	52.8	11,169	35.9	35.2	23.0	63.7
High school	3478	27.6	25.6	16.9	49.4	5070	31.5	34.5	21.9	62.5
University	874	23.5	24.6	16.2	47.0	1288	25.6	25.2	16.9	57.5
Non-smokers	9656	28.3	18.2	13.5	39.2	11,567	30.6	22.8	22.0	45.8
Smokers	4570	29.7	30.9	19.9	55.2	5960	35.7	35.1	22.5	63.8
Non physical activity	7851	50.1	48.5	32.1	83.2	11,899	45.8	50.6	38.9	86.7
Yes physical activity	6375	3.6	6.5	4.6	9.3	5628	3.9	6.9	4.9	11.1
Non Mediterranean diet	8275	47.4	46.0	30.6	78.8	12,536	43.4	48.0	35.6	82.7
Yes Mediterranean diet	5951	4.0	6.9	5.3	10.1	4991	4.5	7.3	5.7	11.7
Non alcohol consumption	8996	13.8	13.5	15.1	12.9	12,332	14.3	15.2	16.2	14.0
Yes alcohol consumption	5230	45.9	49.8	54.6	65.2	5195	52.3	61.3	66.2	72.8

TyG Triglyceride glucose index. BMI Body mass index. METS-IR Metabolic score for insulin resistance. SPISE Single point insulin sensitivity.

**Table 5:** Prevalence of high-risk IR categories in women by sociodemographic and lifestyle factors.

		Non Shift Work					Shift Work			
		TyG High	TyG-BMI High	METS-IR High	SPISE-IR High		TyG High	TyG-BMI High	METS-IR High	SPISE-IR High
Women	*n*	%	%	%	%	*n*	%	%	%	%
18–29 years	1869	4.7	2.9	1.9	7.2	1975	7.1	13.8	7.6	25.7
30–39 years	3103	6.9	6.5	4.5	11.7	3530	9.8	17.1	10.3	30.8
40–49 years	2965	12.5	13.8	8.9	26.0	3450	15.2	20.3	13.0	41.5
50–59 years	1791	24.5	25.8	17.4	45.0	1974	27.0	28.9	18.4	55.6
60–69 years	291	40.5	33.3	23.7	61.5	352	42.9	37.5	26.7	64.8
Social class I	1164	5.6	3.5	2.3	6.2	1644	9.2	10.0	5.2	23.7
Social class II	2763	10.0	7.6	5.4	12.2	4175	13.2	15.0	8.9	32.1
Social class III	6092	14.6	16.0	10.6	30.3	5462	17.9	27.2	17.6	48.0
Elementary school	5377	14.2	16.0	10.4	30.5	4871	17.6	26.4	16.7	47.9
High school	3628	11.4	9.1	6.5	15.4	4984	13.9	17.4	10.8	34.3
University	1014	5.4	3.1	2.2	5.5	1426	9.2	8.8	4.8	22.0
Non-smokers	6638	12.0	8.9	6.1	18.4	7794	14.8	17.0	10.6	36.3
Smokers	3381	12.4	13.9	9.2	24.6	3487	15.1	21.6	13.5	39.6
Non physical activity	4090	23.9	25.3	20.0	54.5	6842	28.6	33.3	22.9	63.3
Yes physical activity	5929	3.6	2.8	2.6	3.8	4439	4.0	3.4	2.7	4.1
Non Mediterranean diet	4206	22.6	29.1	19.5	51.8	7115	26.5	32.1	21.8	60.2
Yes Mediterranean diet	5813	4.1	3.5	3.3	4.2	4166	4.9	4.0	3.4	4.4
Non alcohol consumption	8361	5.8	3.2	1.7	11.0	9619	9.8	13.7	7.6	31.3
Yes alcohol consumption	1658	34.9	38.8	28.9	44.3	1662	44.2	57.9	41.6	80.7

TyG Triglyceride glucose index. BMI Body mass index. METS-IR Metabolic score for insulin resistance. SPISE Single point insulin sensitivity.

**Table 6:** Logistic regression: odds ratios for high-risk IR indices by sex, age, social class, education, lifestyle, and shift work.

	TyG High	TyG-BMI High	METS-IR High	SPISE-IR High
	OR (95% CI)	OR (95% CI)	OR (95% CI)	OR (95% CI)
Women	1	1	1	1
Men	2.34 (2.22–2.47)	1.48 (1.40–1.56)	1.31 (1.23–1.39)	3.54 (3.35–3.74)
18–29 years	1	1	1	1
30–39 years	1.47 (1.32–1.63)	1.10 (1.06–1.15)	1.08 (1.05–1.11)	1.36 (1.27–1.45)
40–49 years	1.84 (1.65–2.04)	1.21 (1.16–1.26)	1.15 (1.10–1.21)	1.43 (1.33–1.53)
50–59 years	2.43 (2.17–2.69)	1.33 (1.26–1.30)	1.20 (1.16–1.25)	1.80 (1.65–1.95)
60–69 years	3.46 (3.03–3.80)	1.44 (1.37–1.51)	1.44 (1.36–1.43)	2.47 (2.11–2.83)
Social class I	1	1	1	1
Social class II	1.51 (1.41–1.62)	1.55 (1.46–1.65)	1.62 (1.49–1.75)	1.51 (1.39–1.63)
Social class III	1.73 (1.60–1.86)	1.65 (1.53–1.78)	1.78 (1.65–1.91)	1.66 (1.53–1.79)
University	1	1	1	1
High school	1.80 (1.68–1.92)	1.43 (1.35–1.52)	1.55 (1.48–1.62)	1.38 (1.30–1.47)
Elementary school	1.53 (1.44–1.62)	1.67 (1.58–1.76)	1.74 (1.63–1.85)	1.60 (1.48–1.72)
Non-smokers	1	1	1	1
Smokers	1.16 (1.12–1.20)	1.19 (1.15–1.24)	1.29 (1.22–1.37)	1.08 (1.05–1.11)
Yes physical activity	1	1	1	1
Non physical activity	10.51 (9.16–11.90)	13.93 (12.13–15.73)	12.81 (11.61–14.03)	16.30 (14.79–17.82)
Yes Mediterranean diet	1	1	1	1
Non Mediterranean diet	1.69 (1.53–1.86)	6.41 (5.50–7.33)	7.86 (6.90–8.84)	2.87 (2.61–3.14)
Non alcohol consumption	1	1	1	1
Yes alcohol consumption	2.45 (2.32–2.58)	5.81 (5.49–6.14)	5.56 (5.22–5.90)	4.62 (4.30–4.95)
Non shift work	1	1	1	1
Yes shift work	1.89 (1.70–2.09)	1.71 (1.62–1.80)	1.49 (1.41–1.58)	1.83 (1.73–1.94)

TyG Triglyceride glucose index. BMI Body mass index. METS-IR Metabolic score for insulin resistance. SPISE Single point insulin sensitivity. OR Odds ratio.

## Data Availability

The dataset generated and analyzed during the current study is securely stored at ADEMA University School in a protected system compliant with all institutional and legal data security standards. The designated Data Protection Officer for the institution is Ángel Arturo López González.
